# Metabolic mechanisms of immunotherapy resistance

**DOI:** 10.37349/etat.2025.1002297

**Published:** 2025-03-13

**Authors:** Luis Cabezón-Gutiérrez, Magda Palka-Kotlowska, Sara Custodio-Cabello, Beatriz Chacón-Ovejero, Vilma Pacheco-Barcia

**Affiliations:** Università degli Studi della Campania “Luigi Vanvitelli”, Italy; IRCCS Istituto Romagnolo per lo Studio dei Tumori (IRST) “Dino Amadori”, Italy; ^1^Medical Oncology, Hospital Universitario De Torrejón, 28850 Madrid, Spain; ^2^Facultad de Medicina, Universidad Francisco de Vitoria, 28223 Madrid, Spain; ^3^Department of Pharmacy and Nutrition, Faculty of Biomedical and Health Sciences, Universidad Europea de Madrid, 28670 Madrid, Spain

**Keywords:** Immune resistance, metabolism, tumor microenvironment, cancer

## Abstract

Immunotherapy has revolutionized cancer treatment, yet its efficacy is frequently compromised by metabolic mechanisms that drive resistance. Understanding how tumor metabolism shapes the immune microenvironment is essential for developing effective therapeutic strategies. This review examines key metabolic pathways influencing immunotherapy resistance, including glucose, lipid, and amino acid metabolism. We discuss their impact on immune cell function and tumor progression, highlighting emerging therapeutic strategies to counteract these effects. Tumor cells undergo metabolic reprogramming to sustain proliferation, altering the availability of essential nutrients and generating toxic byproducts that impair cytotoxic T lymphocytes (CTLs) and natural killer (NK) cell activity. The accumulation of lactate, deregulated lipid metabolism, and amino acid depletion contribute to an immunosuppressive tumor microenvironment (TME). Targeting metabolic pathways, such as inhibiting glycolysis, modulating lipid metabolism, and restoring amino acid balance, has shown promise in enhancing immunotherapy response. Addressing metabolic barriers is crucial to overcoming immunotherapy resistance. Integrating metabolic-targeted therapies with immune checkpoint inhibitors may improve clinical outcomes. Future research should focus on personalized strategies to optimize metabolic interventions and enhance antitumor immunity.

## Introduction

Rapid proliferation and tumor progression constitute one of the hallmarks of cancer. Metabolism convert nutrients into metabolites by which cells generate macromolecules (lipids, DNA, proteins, RNA), redox equivalents and energy that cells need to sustain their functions [1]. Tumor cells, have an urgent need for proliferation and, therefore, require a more efficient and faster metabolic pattern than normal cells: the “Warburg effect” or aerobic glycolysis [2]. In this context, the ATP production is more efficient than in the oxidative phosphorylation (OXPHOS) but the ATP production per molecule of glucose is lower [3, 4]. Tumor cells can alter metabolic pathways and can also suppress anti-tumor immune response [5, 6] in order to increase the nutrients available into biomass energy. In the tumor microenvironment (TME), cancer cells have a metabolic reprogramming [7] to ensure the energy-demanding anabolic requirements for tumor progression and cell proliferation [8]. Furthermore, metabolic reprogramming has also been observed in the TME [2].

The extracellular matrix (ECM), blood and lymphatic tumor vessels and cancer-associated fibroblasts (CAFs) are part of the TME and modulate cancer cell proliferation and the resistance of systemic therapies [3]. The TME promote growth and invasion of tumor cells via abnormal angiogenesis and with irregular extracellular matrices like cytokines [6, 9]. Tumor-immune landscapes may be altered by the lack of nutrients and the accumulation of metabolic intermediates that can have an impact in the anti-tumor functions of immune cells [10, 11]. It is well-known that the immune system plays an important role in the interplay of cancer and can suppress cancer progression [12]. Accumulating evidence suggests that the immune response may be altered by the dysregulation of tissue metabolism, mostly associated with the lack of nutrients, the increased oxygen consumption and the metabolic potentially toxic intermediates of reactive nitrogen and oxygen [9, 13, 14] and it has been observed that immune cells compete with cancer cells in the TME for nutrients [15].

The balance of OXPHOS and glycolysis is an important aspect of metabolic reprogramming [16]. Metastatic tumors can harbor a predominant complex metabolic phenotype including OXPHOS to provide higher energy efficiency and support more invasion and growth. In the TME, T cells lose their ability to address cancer by T cell exhaustion which is a consequence of changes in RNA metabolism [17]. In addition, immune cells such as dendritic cells, T cells and immune cells suffer from metabolic reprogramming in the TME and changes in the nucleotide metabolism contribute to the dysfunction of these cells. The metabolic alterations of cancer cells can diminish the effectiveness of immunotherapies because of metabolic rewiring that suppresses immune responses and, ultimately, contribute to immunotherapy resistance [18].

In this review, we analyze the role of metabolic reprogramming in the TME and the impact it may have in therapeutic strategies.

## Impact of dysregulated glucose metabolism in TME

Dysregulation of the glucose metabolism in TME can associate alterations in intermediate metabolites and several enzymes [10]. As previously mentioned, glycolysis may be accelerated by tumor cells independently of the oxygen that is available as explained for the Warburg effect [19]. The hallmarks of dysregulated glucose metabolisms are an increase in glycolysis, decrease in gluconeogenesis and metabolic intermediates, like lactate and itaconate, that ultimately suppress immunosurveillance and have an unfavorable effect on TME [10]. Furthermore, these changes suppress cytotoxicity of cytotoxic T lymphocytes (CTLs), macrophages and natural killer (NK) cells while increasing immunosuppression mediated by myeloid-derived suppressor cells (MDSCs), M2 polarization and Tregs. Metabolic alterations can be influenced by cancer stage: 1. early-stage tumors can harbor localized metabolic rewiring; 2. advanced-stage tumors can present aggressive metabolic changes to support progression, metastastases and tumor survival in distant tissues.

### Lactate accumulation

Cancer cells produce lactic acid in large amounts with the consumption of glucose, even though there is enough oxygen, and it accumulates in cells prior transportation into the extracellular environment where it ultimately establishes and acidic TME [20]. A more aggressive type of cancer cells have been observed in acidic TME and it can also promote cancer progression by suppressing immune anti-tumor response [21]. This sums up with the fact that lactic acid levels increase lineally with the tumor burden [22] and it can be used as a nutrient source by cancer cells [23]. Lactic acid in the TME can suppress cancer immunosurveillance mechanisms and promote immune escape of cancer cells: 1. lactic acid affects NK cells and reduces interferon gamma (IFN-γ) secretion promoting apoptosis [24, 25]; 2. lactate in the TME activates the phosphorylation the transcription factor via mTOR pathway inhibiting vacuolar ATPase subunit in macrophages [26]; 3. lactate concentration in the TME has shown a negative correlation in survival of patients with cervical cancer [27].

When there is a fast oxygen consumption and an altered angiogenesis the hypoxia-inducible factor (HIF) is activated by hypoxia and it upregulates programmed death-ligand 1 (PD-L1) expression [10]. The accumulation of lactate interrupts lactate homeostasis by the influence of HIF-1, c-myc and PI3K/AKT oncogenes [28]. T-cell exhaustion occurs by the PD-1/PD-L1 pathway when the IFN-γ secretion is increased by lactate levels [10]. In addition, lactate blocks the transporter MCT-1 at the same time as it enhances Tregs by nuclear factor of activated T-cells 1 (NFAT1) translocation into nucleus and increases lactated absorption by upregulating MCT-1 and the expression of lactate dehydrogenase A which in all disrupts the anti-tumor effects of CTLs [29]. Tregs suppress CD8^+^ T-cells function of secreting perforin and cytotoxic granzymes. The tricarboxylic acid (TCA) cycle is also supported by lactate when there is an increase in IL-10 and TGF-β which enables proliferation in acidic and hypoglycemic conditions [30, 31]. Anti-tumor responses are also negatively influenced by the enhancement of M2 polarization by inducing arginase-1 (ARG-1), transcriptional repressors, HIF-1α and IL-6 via the upregulation of MCT-4 expression and glycolytic rate promoted by lactate [32]. The impact of lactate on TME tumor evasion has also been observed with HLA-DR- and CD86-high macrophages have a glycolytic phenotype that suppresses the secretion of anti-tumor IL-12 p70 via PKM2/HIF-1α axis [33, 34].

OXPHOS takes place in mitochondria to efficiently produce ATP with substrates like glucose, fatty acids (FAs) and amino acids. Cancer cells can prefer OXPHOS to generate ATP in less glucose-rich or more oxygenated tissues in order to support redox balance and biosynthesis. In more aggressive, rapidly growing tumors or hypoxic regions, tumor cells may rely on glycolysis. In well-vascularized regions with oxygen they can use OXPHOS, sometimes both mechanisms coexist and it can vary across tumor types [35].

### Itaconate

Itaconate is synthesized from the decarboxylation of TCA-cycle derived *cis*-aconitate regulated by the mitochondrial enzyme immune-responsive gene 1 (IRG1) [36, 37]. Itaconate induces CD8^+^ T exhaustion, attenuate CD8^+^ T-cell proliferation and function and can suppress myeloid immune cells [36]. Itaconate could be a potential immunometabolite target in the TME [37, 38]: 1. high levels of IRG1 and itaconate increases ROS generation in macrophages via MAPK pathway; 2. inhibition of succinate dehydrogenase (SDH), glycolysis mediation, Nrf2 transcription factors and ATF3 activation associated with the modulation of exhaustion markers TIM-3 and PD-1 [37, 38].

## Impact of dysregulated lipid metabolism in TME

In the context of a non-sufficient nutrient supply, there is a change of metabolism: from glucose-dependent to lipid-dependent. On the one hand, lipids are a potential energy source for growth and proliferation, energy and membrane formation. On the other hand, lipids mediate post-translational modifications to regulate HIF-1α, nuclear factor kappa-light-chain-enhancer of activated B cells (NF-κB), STAT3 and AP-1 and TGF-β and Wnt pathways [39].

### PGE2

Arachidonic acid is a class of eicosapentaenoic acid and it is a substrate for prostaglandins synthesis associated with FAs in tumor cells [12]. PGE2 is an immunosuppressive factor [40] necessary for cell growth as well as a regulation factor in the cell that mediates inflammatory response [41]. PGE2 could play an active role in tumor progression by promoting cancer cell invasion, stimulating angiogenesis and suppressing apoptosis which ultimately enhances cell proliferation [42]. The immune response could also be altered by PGE2 by autocrine and paracrine methods. PGE2 accumulation can increase M2 macrophages that induce cancer by transforming M1 macrophages, that harbor a tumor suppressing effect [43]. Anti-inflammatory effects of PGE2 have also been observed in NK cells, neutrophils and monocytes [44, 45]. Furthermore, PGE2 can promote immune scape mechanisms and tumorigenesis: 1. it can stimulate bone marrow-like cells to promote CXCL1, IL-6 and granulocyte-colony-stimulating factor (G-CSF); 2. inhibit the production of IL-12 and TNF-α in myelocid cells stimulated by lipopolysaccharide (LPS).

In the TME, PGE2 can promote an immunosuppressive state by both preventing the pro-inflammatory response in immune cells, reprogram TME and also by directly upregulating tumor cells [46].

### Fatty acids

Plasma membrane phospholipids and molecules for signaling are generated via the anabolic pathways used by tumor cells which require an increase in the synthesis of de novo FAs [12]. On the other hand, immune cells require FAs for cell membranes and lipidic cell structures [47]: 1. effector immune cells that harbor a rapid growth require FAs for cell membranes; 2. memory immune cells grow slower and doesn’t need that amount of biosynthesis so the main way is by the FA oxidation (FAO) [48]. The metabolic reprogramming associated with the lipid metabolism can lead to an immunosuppressive state by the abnormal accumulation of short-chain FA, cholesterol, long chain FA, etc., in myeloid cells that are tumor-infiltrating [49].

The impact of FA on T-cells depends on the amount of FA: low concentration of FA can impact proliferation while an increase in FA concentration can lead to apoptosis because of lipotoxicity [50] and, therefore, FA catabolism can upregulate immune response. In the TME, dysregulated FAO and the modulated carnitine palmitoyl-transferase (CPT), that transports acyl-CoA into mitochondria, in cells act directly in the metabolic remodeling [51]. The receptor-interacting protein kinase 3 (RIPK3)-ROS-Caspase-PPAR pathway promotes FAO and M2 polarization of tumor-associated macrophages (TAMs): 1. inositol-requiring protein 1α (IRE-1α)-mediated endoplasmic reticulum (ER) stress which obtains immune inefficiency in DC and M2 polarization by inducing lipid peroxidation; 2. Tregs have higher FAS and FAO while TILs evade oxidative stress and show T-cell disability [52, 53]; 3. RIPK3 exacerbates M2 polarization and is downregulated in TAMs; 4. the shortage of RIPK3 is associated with decreased ROS and the inhibition of caspase-1mediated PPAR to ultimately promote FA metabolism [54].

In metastatic cells, FAO provides acetyl-CoA and are a substrate for OXPHOS, important to contribute to the high energy demand [55]. In organs where metabolic conditions favor the use of lipids, like liver and bone, the reliance of FAO is higher [56]. FAs synthesis plays an important role in cancer to induce energy production, membrane biosynthesis and are also involved in signaling molecules. Tumors such as prostate, ovarian, breast, colorectal and lung cancer are dependent on FA biosynthesis.

### Cholesterol

Cholesterol is a part of the cell membrane and tumor cells with rapid proliferation mechanisms require more synthesis. Tumor cells that have a high expression of cholesterol can evade immune surveillance mechanisms and treatment strategies [57]. High levels of cholesterol in immune cells have been associated with an increase in the expression of immune detection points as LAG-3, TIM-3 and PD-1, however, researchers have observed that there is more concentration of cholesterol in tumor cells compared to the levels in immune cells [58]. In this context, high concentrations of cholesterol can downregulate anti-tumor effect of T cells [59].

The autoxidation of cholesterols results in metabolites called oxysterols, always associated with high excess of cholesterols. Oxysterols have functions that can alter the immune response and landscape. The 22-hydroxycholesterol (22-OHC) can engage CD11b^high^ and Gr1^high^ neutrophils in cancer cells and act as an immunosuppressor in the TME [60]. The 27-hydroxycholesterol (27-OHC) has been described in high concentrations in tumors of patients with estrogen receptor-positive breast cancer [61]. 27-OHC has been associated with CD8 T cells exhaustion that can promote tumor proliferation and metastasis [62]. Previous studies point out that lowering cholesterol concentration in the TME can alleviate T cell immune suppression [12].

## Impact of dysregulated amino acid metabolism in TME

Amino acids participate in the synthesis of proteins and nucleotids and plays a crucial role in cancer metabolism.

### Nucleic acid metabolism

Cancer cells have a rapid and non-controlled proliferation that requires an active nucleic acid metabolism in the TME [63]. The dysregulated nucleic acid metabolism collaborates with cell division promotion, genetic integrity and adaptation to hostile conditions. Cancer cells have a DNA and RNA altered metabolism that affects the ability of tumor cells to grow, metastasize and, ultimately, survive. Due to the increased demand for nucleids, cancer cells overexpress purine and pyrimidine biosynthesis pathways to increase nucleotide synthesis. Therefore, enzymes like thymidylate synthase, inosine monophosphatase dehydrogenase and AMP deaminase are upregulated to supply nucleotide synthesis.

In DNA replication, cancer cells upregulate and enhance the availability of nucleotides for synthesis. In DNA repair, pathways such as homologous recombination, nucleotide excision repair and base excision repair are increased although a dysregulated repair can contribute to resistance to therapy and genomic instability [64]. RNA metabolism often requires alterations in RNA polymerase activity to support the uncontrolled cell division and adapt to hypoxia and nutrient depravation. In this context, RNA-binding proteins or microRNA profiles can be altered to regulate gene expression [65]. Furthermore, alternative splicing can change proteins to evade immune surveillance, promote tumorigenesis and contribute to metastasis.

### Glutamine

Glutamine is a key component of amino acid production, glycosylation, autophaghy, epigenetics, the synthesis of nucleotides and ECM production [66], therefore, it is important for the proliferation of both cancer cells and normal cells [67]. Macrophages, lymphocytes and neutrophils require glutamine for the development of cell fate and immune responses [68, 69]. The transformation of CD4^+^ T cells to inflammatory subtypes is upregulated by glutamine metabolism as well as the activation of immune cells [69, 70]. Glutamine is also required for macrophages to produce and secrete pro-inflammatory cytokines (IL-1, IL-6 and TNF) [12]. If there is a nutritional deficiency, cancer cells are able to destroy large molecules in order to obtain glutamine as it has been observed in the excessive activation of the oncogene RAS where tumor cells degrade extracellular proteins into amino acids which include glutamine in order to supply and provide more nutrients to maintain cancer cell proliferation [71]. Glutamine also plays an important role in fueling OXPHOS in metastatic tumor when glutamine is converted to glutamate and further alfa-ketoglutarate that enters the TCA cycle and sustains OXPHOS [72].

### Arginine

The argininosuccinate synthethase 1 (ASS1) is an enzyme envolved in the production of arginine and is lacking in the majority of cancer cells which lowers the capacity of synthetizing arginine and, therefore, tumor cells will use exogenous arginine [73]. As previously mentioned for glutamine, arginine also plays an important role in immune responses and T cell activation [12]: 1. arginine stimulates T cell and NK cell cytotoxicity as well as effector cytokine production; 2. stimulation of effector cytokine production in vitro and in treatment with anti-PD-L1, mice with osteosarcoma showed longer survival rates [74]. In previous studies, immunomodulatory cells that express ARG-1 (M2-TAMs, Treg cells and tolerogenic DCs) can increase the degradation of arginine and limit the source for T cells [75].

### Tryptophan

Tryptophan is an essential amino acid required for protein synthesis and metabolism and it can degradate by inoleamine-2, 3-dioxygenase (IDO1) and tryptophan-2, 3-dioxygenase (TDO2) to tryptophan to kynurenic acid [76]. Previous studies have determined that in gastric adenocarcinoma patients who harbor high levels of IDO1 and TDO2, these enzymes can promote tumor progression and are associated with a worse prognosis [77]. The immune activity and function of T cells can be suppressed if there is low availability of tryptophan in the TME and it has been suggested that high levels of IDO1 and TDO2 in the TME are associated with lower values of tryptophan [12]. As it occurs for arginine, when tumor cells use tryptophan, it consequently associates a lack of tryptophan for T cells and it can trigger apoptosis in these immune cells [78, 79]. In the presence of an IDO1 inhibitor, tryptophan levels can be increased and the accumulation can induce tumor regression by upregulating the production of IL-12 and IFN-γ cytokines and T cells and neutrophils in mouse metastastic liver and blade tumor models [12].

## Local metabolic regulation

The TME is a dynamic and complex network surrounding cancer cells, comprising various cellular and noncellular elements that critically influence cancer progression, metastasis, and resistance to therapies [80]. Key cellular components include stromal fibroblasts, immune cells such as macrophages and lymphocytes, endothelial cells forming blood vessels, and the ECM [81]. These elements interact with cancer cells via biochemical and mechanical signals, creating a niche that fosters tumor growth and impedes therapeutic efficacy [82].

### CAFs and ECM remodeling

CAFs are key regulators of the TME through the secretion of growth factors, cytokines, and ECM proteins that facilitate tumor proliferation, invasion, and angiogenesis [83]. In addition to providing structural support for tumor expansion, CAFs actively remodel the ECM, creating physical barriers that limit immune cell infiltration [84].

### Hypoxia and metabolic reprogramming in the TME

Hypoxia, a hallmark of solid tumors resulting from aberrant vasculature, exacerbates the complexity of the TME. Under hypoxic conditions, the stabilization of HIFs promotes angiogenesis, metabolic reprogramming, and epithelial-to-mesenchymal transition (EMT), thereby enhancing metastatic potential [85–87]. Moreover, the acidic and nutrient-depleted nature of the TME, driven by altered cancer cell metabolism, influences both stromal and immune cell function. The Warburg effect, characterized by increased aerobic glycolysis and lactate production, contributes to an immunosuppressive and acidic microenvironment that further supports tumor progression and immune evasion [88, 89].

### TAMs and immunosuppression

TAMs are abundantly present in the TME and play a central role in tumor progression [90]. Chemokines such as colony-stimulating factor 1 (CSF1) and C-C motif chemokine ligand 2 (CCL2) recruit monocytes from peripheral circulation, where they differentiate into macrophages [91]. These macrophages can adopt either an M1 phenotype, which exhibits tumoricidal activity, or an M2 phenotype, which promotes tumor growth, metastasis, and immune suppression through inhibition of CD8^+^ T cells [91].

### The impact of glucose metabolism on TAM polarization

Glucose metabolism significantly influences TAM polarization toward a tumor-promoting state. While tumor cells are known to produce lactate via glycolysis, recent findings suggest that TAMs exhibit an enhanced capacity for glucose consumption and lactate production, thereby contributing to the acidic TME [92]. Lactate has been shown to drive TAM polarization toward the M2 phenotype, leading to increased expression of vascular endothelial growth factor (VEGF) and ARG-1. VEGF facilitates angiogenesis, whereas ARG-1 catalyzes polyamine synthesis, supporting cancer cell proliferation [93]. Furthermore, lactate suppresses ATP6V0d2 expression in macrophages, preventing lysosomal degradation of HIF-2α, thereby further promoting tumor progression [94, 95]. Additionally, lactate enhances PD-L1 expression through NF-κB, reinforcing TAM-mediated immunosuppression [96].

### Amino acid and lipid metabolism in TAM-mediated tumor support

The tumor-supportive function of TAMs is further driven by amino acid and lipid metabolism. Methionine adenosyltransferase II α (MAT2A) elevates S-adenosylmethionine (SAM) levels in macrophages, facilitating TAM polarization via histone methylation [97]. Additionally, increased expression of lipid metabolism genes in TAMs highlights their metabolic adaptation within the tumor niche [98]. Notably, downregulation of monoacylglycerol lipase (MGLL) in TAMs supports tumor growth by altering the catabolism of 2-arachidonoylglycerol (2-AG), which activates cannabinoid receptor-2 (CB-2) and promotes M2 macrophage polarization [99].

### Organ-specific metabolic features of tumors

The anatomical location of the tumor also influences its metabolic characteristics. Tumors exhibit organ-specific metabolic profiles shaped by local nutrient and oxygen availability, which significantly impact the behavior of tumor and immune cells [100, 101]. For example, liver tumors have distinct metabolic adaptations compared to brain tumors, highlighting the role of the local microenvironment in cancer progression.

In colorectal cancer, metabolic reprogramming leads to altered nutrient availability and a greater reliance on glycolysis, driving lactate accumulation and reinforcing immune suppression [102, 103]. These metabolic adaptations within the TME underscore their role in both cancer progression and resistance to immunotherapy. Consequently, targeting the metabolic flexibility of tumor cells and their interactions within the TME offers a promising avenue for improving therapeutic outcomes.

**Figure 1:**
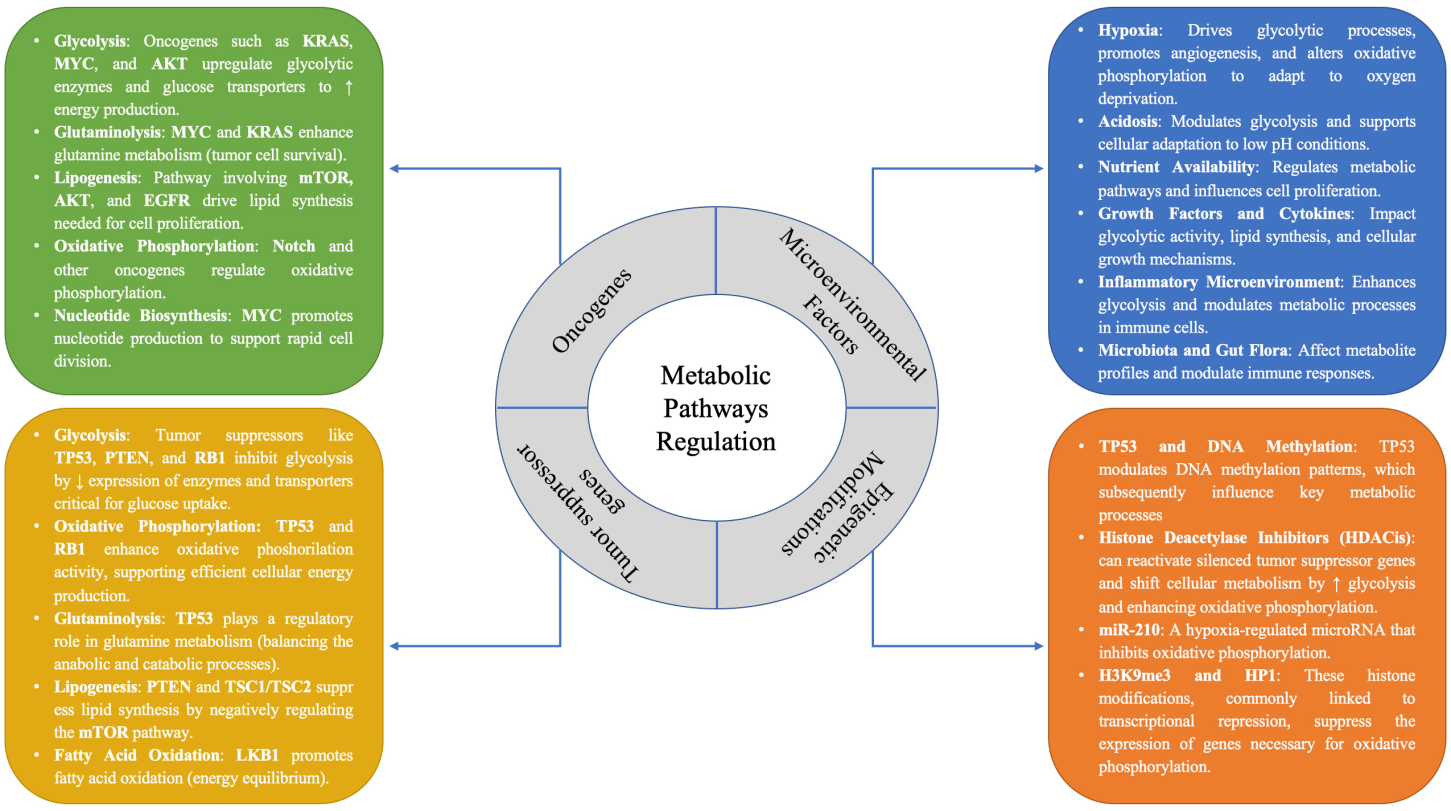
The regulatory framework of metabolic pathways in cancer, emphasizing the pivotal roles of oncogenes and tumor suppressor genes in modulating these processes

The impact of these metabolic alterations on the regulation of oncogenes, tumor suppressor genes, epigenetics, and microenvironmental factors is summarized in Figure 1.

## Targeting metabolism to restore immunity

Since the Warburg effect was first described in the 1920s [104], the distinct metabolic profile of cancer cells has drawn significant attention, with a focus on how these metabolic processes can be exploited to enhance cancer treatment. Immunotherapy strategies, such as immune checkpoint inhibitors (ICIs), virotherapy, vaccines, and adoptive cell therapy (ACT), aim to boost the immune response against tumors. Nevertheless, response rates to immunotherapy remain suboptimal, partly due to intratumoral heterogeneity and variations in metabolic pathways, as discussed in previous sections. Tumor metabolism is pivotal in shaping tumor immunity and influencing the effectiveness of immunotherapy. The high demand for nutrients by cancer cells to support their rapid proliferation limits the availability of metabolic resources for immune cells, thereby reducing immune activity [105]. The metabolic activity of cancer cells suppresses T cell metabolism and immune function. Tumor cells with elevated glycolytic activity impair T cell growth by depleting glucose within the TME, hindering T cell-mediated cytokine production.

This section highlights the latest findings on potential strategies, primarily in preclinical stages, aimed at modulating immunosuppressive TMEs, enhancing immune responses, and overcoming immune checkpoint resistance through metabolic targeting.

Since a high-lactate microenvironment suppresses antitumor immunity and facilitates tumor progression [106], reducing lactate production has emerged as a strategy to improve immunotherapy outcomes. One of the initial approaches involved neutralizing lactate directly within the TME using alkaline agents, which demonstrated a significant enhancement in the efficacy of ICIs [107]. Additionally, studies by Renner et al. [108] showed that inhibiting monocarboxylate transporters in cell and animal models markedly increased the therapeutic effects of PD-1 and CTLA-4 inhibitors.

Tumors achieve an alkaline intracellular pH while creating an acidic extracellular environment through the activity of the Na/H exchanger isoform 1 (NHE1) [89]. Blocking NHE1 has been shown to inhibit tumor growth, improve survival, and enhance cytotoxic T cell infiltration. In a mouse model of glioma, combining NHE1 inhibition with anti-PD-1 antibodies and temozolomide treatment resulted in a significant extension of survival, indicating that altering the pH of the TME could boost T cell activity [109].

Lactate, which is produced by various cells in the TME such as fibroblasts and tumor cells, contributes to TME acidification. This accumulation of lactate suppresses the transcription of the FAK family interacting protein of 200 kDa (FIP200) by lowering NAD levels [110]. The absence of FIP200 in T cells leads to apoptosis in naive T cells, thereby impairing antitumor immune responses. Additionally, lactate promotes the sirtuin 1 (SIRT1)-mediated degradation of T-bet and increases the presence of Tregs, which correlates with heightened aggressiveness in prostate cancer [111].

Beyond lactate, a growing number of tumor-derived metabolites contribute to T cell dysfunction and resistance to ICI within the TME. Mutations in SDH result in the accumulation of succinate in tumor cells, which impairs T cell IFN-γ secretion by disrupting glucose metabolism in the TCA cycle [112]. Therefore, targeting succinate to lower its levels pharmacologically may help to restore antitumor immune activity.

In colorectal cancer, elevated ammonia levels have been observed, leading to metabolic reprogramming of T cells and promoting T cell exhaustion. Consistently, cancer patients present increased serum ammonia, with ammonia-related gene expression signatures correlating with T cell responses and ICI efficacy. In mouse models of both primary and metastatic colorectal cancer, enhancing ammonia clearance has been shown to activate T cells, inhibit tumor growth, and improve tumor sensitivity to ICIs [113].

The interaction between HIF-1α and histone deacetylase 1 (HDAC1) contributes to T cell dysfunction through chromatin remodeling, resulting in the epigenetic suppression of T cell effector gene expression. Targeting HIF-1α inhibition has been shown to restore T cell function and overcome resistance to ICI in both syngeneic and humanized mouse models of triple-negative breast cancer [114]. Moreover, the use of a hypoxia-targeted chemotherapeutic prodrug in combination with ICI has been effective in sensitizing prostate cancer to ICI treatment. This approach reduces myeloid cell-mediated suppression and enhances the infiltration of effector T cells, leading to sustained tumor remission [115, 116]. These findings suggest that targeting hypoxia-related pathways may improve tumor sensitivity to ICI.

The metabolic landscape of the TME also contributes to ICI resistance by depleting essential nutrients. Both immune cells and tumor cells rely on critical resources like glucose and amino acids to support their bioenergetic, biosynthetic, and redox needs. However, tumors often outcompete immune cells for these nutrients, leading to nutrient scarcity that can weaken antitumor immune responses and foster ICI resistance. For instance, tumor cells interfere with methionine metabolism in CD8^+^ T cells, resulting in lower levels of SAM. This alteration disrupts demethylation at histone H3 lysine 79 (H3K79), thereby impairing STAT5 signaling and T cell functionality, which can contribute to ICI resistance [117]. Studies have shown that methionine supplementation can enhance T cell responses in tumor-bearing mice and colorectal cancer patients [97]. Similarly, arginine is vital for T cell activity within the TME. Elevating arginine levels shifts T cells towards OXPHOS, thereby boosting their antitumor capacity [118]. Conversely, a reduction in arginine may impair T cell function and promote resistance to ICI therapy.

The glutamine antagonist 6-diazo-5-oxo-*L*-norleucine has been shown to inhibit the glycolytic activity of cancer cells while simultaneously promoting oxidative metabolism in T cells [119]. This distinct metabolic response to glutamine inhibition between cancer cells and T cells highlights an important metabolic divergence, which could serve as a valuable therapeutic target in cancer patients [118]. With regard to glutamine metabolism, various drugs have been developed, showing promising preliminary results in animal studies. The primary goal has been to limit glutamine uptake by cancer cells. For instance, Byun et al. [120] reported that combining glutamine transporter inhibitors with PD-L1 blockers significantly boosted the effectiveness of ICIs in lung and colon cancer models. Their research also indicated that PD-L1 expression restricted glutamine utilization in T cells. Similarly, some agents target glutaminase (GLS) to inhibit intracellular glutamine metabolism, such as JHU083 and CB839. In colon cancer-bearing mice, PD-1 inhibition alone had minimal impact, whereas combining it with the GLS inhibitor JHU083 led to nearly a 100% improvement in antitumor activity [119]. The experimental GLS inhibitor CB839 also showed potential in enhancing CAR-T cell accumulation within tumor tissues [69].

The regulation of cystine levels within the TME in cancer patients remains largely unclear. Evidence suggests that IFN-γ secreted by T cells can decrease the expression of cystine-glutamate transporters SLC3A2 and SLC7A11, leading to reduced intracellular levels of cystine, cysteine, and glutathione. This depletion facilitates tumor cell lipid peroxidation and induces ferroptosis. Moreover, the reduction of cystine via cystinase has been found to enhance antitumor immunity and improve the effectiveness of ICB in mouse models with tumors [121].

Cholesterol secreted by tumors has been found to hinder antitumor immune responses. Within the TME, elevated cholesterol levels upregulate CD36 expression in infiltrating CD8^+^ T cells, leading to increased FA uptake and subsequent lipid peroxidation and ferroptosis, which ultimately compromise T cell-mediated immunity [122]. Additionally, cholesterol contributes to immune checkpoint expression and T cell exhaustion by triggering ER stress that modulates PD-1 levels on tumor-infiltrating CD8^+^ T cells [123]. Blocking cholesterol esterification in T cells has been shown to enhance their proliferation and effector capabilities [104]. However, because cholesterol is essential for T cell receptor signaling through membrane clustering, altering cholesterol metabolism to bolster the antitumor response to ICI presents significant clinical challenges [124]. Other lipid species, including arachidonic acid, palmitoleic acid, and oleic acid, have demonstrated potential in promoting tumor ferroptosis in response to IFN-γ and could act synergistically with ICI in murine studies [125]. Despite these findings, research on the role of various lipids in tumor immunity and ICI remains limited.

Beyond amino acids and lipids, glycolytic reprogramming also plays a critical role in shaping tumor immunity and influencing the success of ICI. Activated T cells rely heavily on aerobic glycolysis for effective antitumor activity [126, 127]. Tumor and myeloid cells compete with T cells for glucose in the TME, which impairs T cell function by suppressing Notch signaling and reducing mTOR activity and IFN-γ production, thus enabling tumor growth [15, 128]. Notably, increased glycolytic activity in tumors can override T cell-mediated tumor suppression. The glycolytic intermediate phosphoenolpyruvate is crucial for sustaining NFAT signaling and T cell effector functions, and its enhancement in the TME has been shown to boost T cell activity [129]. This highlights how tumors manipulate fundamental metabolic pathways in immune cells to evade immune responses and foster resistance to ICI therapy. OXPHOS activity is associated with an increased resistance to immune response and tumors minimize immunogenic metabolic production, such as lactate, diminishing immune responses [130]. Furthermore, tumors with OXPHOS activity and mitochondrial function can reduce pro-inflammatory signals and immune-stimulating metabolites [131].

This review includes a summary of the mechanisms of these drugs and their impact on cancer immunotherapy, as detailed in Table 1.

**Table 1:** Drugs for targeted metabolism combined with immunotherapy

**Drug**	**Targeted metabolism**	**Mechanism**	**Appropriate immunotherapy**	**Reference**
Bicarbonate	Glycolysis	Directly increase pH value	Anti-PD-1/L1/CTLA-4 treatment	[107]
Diclofenac	Glycolysis	Inhibit lactate transporter protein	Anti-PD-1/L1 treatment	[108]
CB839	Glutaminolysis	Inhibit GLS activity	CAR-T cell therapy	[69]
JHU083	Glutaminolysis	Inhibit GLS activity	Anti-PD-1/L1 treatment	[119]
V-9302	Glutaminolysis	Inhibit glutamine transporter protein	Anti-PD-1/L1 treatment	[120]
Glycolytic metabolite phosphoenolpyruvate (PEP)	Glycolysis	Sustaining NFAT signaling and T cell effector functions, and its enhancement in the TME has been shown to boost T cell activity	Anti-PD-1/L1 treatment	[109]
Methionine supplementation	Methionine metabolism	Improved the expression of H3K79me2 and STAT5 in T cells, and this was accompanied by increased T cell immunity	Anti-PD-1/L1 treatment	[117]
Chemotherapeutic prodrug (TH-302)	Hypoxia-inducible factor	This approach reduces myeloid cell-mediated suppression and enhances the infiltration of effector T cells	Anti-PD-1/L1 treatment	[116]
Arachidonic acid	Lipid metabolism	IFN-γ in combination with arachidonic acid induces immunogenic tumor ferroptosis, serving as a mode of action for CD8^+^ T cell mediated tumor killing	Anti-PD-1/L1 treatment	[125]
Avasimibe	Lipid metabolism	Inhibition of ACAT1, a crucial enzyme involved in cholesterol esterification, resulted in enhanced effector functions and increased proliferation of CD8^+^ T cells, while having no significant effect on CD4^+^ T cells	Anti-PD-1/L1 treatment	[124]

GLS: glutaminase; IFN-γ: interferon gamma; ACAT1: acyl-CoA acyltransferase 1; NFAT: nuclear factor of activated T-cells; TME: tumor microenvironment

### Future research directions

Despite the promising potential of targeting tumor metabolism to overcome immunotherapy resistance, several challenges remain to be addressed. Tumor metabolic heterogeneity poses a significant barrier, as different tumors and even different regions within the same tumor may exhibit distinct metabolic profiles [47]. This heterogeneity complicates the development of universal metabolic-targeting therapies and necessitates personalized approaches based on tumor-specific metabolic signatures.

Many drugs that work in preclinical models have been evaluated, but unfortunately, they do not work for patients. Below, we summarize some of them and discuss the reasons why they may not have worked to date. Table 2 summarizes them.

**Table 2:** Clinical trials with drug targeting metabolic reprogramming

**Metabolic target**	**Experimental drug**	**Combination drug**	**Cancer type**	**Reference**
Anti-mitochondrial (tricarboxylic acid cycle)	Devimistat	Chemotherapy	Pancreatic cancer	[132]
Glutaminase inhibitor	Telaglenastat	Cabozantinib	Renal clear-cell cancer	[133]
IDO1	Epacadostat	Pembrolizumab	Melanoma	[134]
Pegylated recombinant human hyaluronidase	Pegvorhyaluronidase alfa (PEGPH20)	Chemotherapy	Pancreatic cancer	[139]
OXPHOS inhibitor	IACS-010759	None	Myeloid leukemia and advanced solid tumors	[140]
Arginase inhibitor	INCB001158	Retifanlimab	Advanced solid tumors	[142]

IDO1: inoleamine-2, 3-dioxygenase; OXPHOS: oxidative phosphorylation

Devimistat, an investigational anti-mitochondrial agent, targets cancer cell mitochondria by disrupting energy production, specifically the TCA cycle. Its efficacy was evaluated in the AVENGER 500 trial (ClinicalTrials.gov identifier: NCT03504423), a global, randomized phase III study [132]. The trial assessed the efficacy and safety of devimistat combined with modified FOLFIRINOX versus standard-dose FOLFIRINOX in treatment-naive patients with metastatic pancreatic cancer (mPC). The primary endpoints were overall survival (OS) and progression-free survival (PFS), with no significant differences observed between the control and experimental arms. No new toxicity signals were observed. These findings indicate that devimistat, when combined with chemotherapy, did not improve long- or short-term outcomes in mPC patients compared to standard chemotherapy. The lack of efficacy of devimistat may be attributed to several factors. Pancreatic cancer (PC) exhibits prognostically distinct metabolic subgroups based on glycolytic and cholesterogenic gene expression. Tumors with poor prognosis often show low expression of mitochondrial pyruvate carriers MPC1 and MPC2, potentially reducing the effectiveness of metabolism-targeting agents such as devimistat. However, due to resource limitations, the AVENGER 500 protocol did not mandate baseline tumor tissue collection for gene expression analysis, leaving potential imbalances in metabolic or basal subtypes across treatment arms undetermined. Additionally, the dosing and administration schedule used in the study may have contributed to the lack of observed efficacy.

Telaglenastat is an investigational, first-in-class, selective, oral GLS inhibitor that blocks glutamine utilization and downstream pathways. Preclinically, telaglenastat synergized with cabozantinib, a VEGFR2/MET/AXL inhibitor, in renal clear-cell cancer (RCC) models. CANTATA (ClinicalTrials.gov Identifier: NCT03428217) was a randomized, placebo-controlled, double-blind, pivotal trial in patients had metastatic RCC following progression on 1 to 2 prior lines of therapy [133]. Compare the efficacy and safety of telaglenastat plus cabozantinib versus placebo plus cabozantinib. The primary endpoint was PFS assessed by blinded independent radiology review. No differences were observed in terms of PFS, objective response rate (ORR), or grade 3/4 adverse events between the experimental arm and the control arm. Possible reasons for the lack of efficacy of telaglenastat in the CANTATA trial include insufficient drug exposure, although pharmacokinetic analyses suggest otherwise. Cabozantinib may not be the optimal partner for dual metabolic inhibition of glucose and glutamine. Additionally, telaglenastat may not sufficiently inhibit GLS in vivo, or GLS may not be an ideal target in RCC at the tested dose. Identifying biomarkers of response could clarify these mechanisms.

Epacadostat, an IDO1 selective inhibitor, was evaluated in a randomized, placebo-controlled, double-blind, phase 3 trial (ClinicalTrials.gov Identifier: NCT02752074) in patients with unresectable stage III or IV melanoma previously untreated with PD-1 or PD-L1 inhibitors [134]. Primary endpoints were PFS and OS. Epacadostat plus pembrolizumab did not improve PFS or OS compared to placebo plus pembrolizumab. No relevant differences were observed in grade 3/4 adverse events. The absence of benefit of adding epacadostat to pembrolizumab was surprising, given early-phase clinical data. In two open-label, phase 1–2 studies of patients with advanced melanoma, the combinations of epacadostat plus pembrolizumab (ECHO-202) [135] and epacadostat plus nivolumab (ECHO-204) [136] showed promising antitumour activity (56–65% of patients achieving an ORR) compared with historical data. Epacadostat was chosen based on preclinical and clinical data showing optimal activity and tolerability. However, higher doses might be needed for better target coverage. The role of IDO1 in melanoma is controversial due to variability in IDO expression. The high response rates and favorable PFS in early trials remain unexplained, despite similar patient and tumor characteristics to other studies.

Pegvorhyaluronidase alfa (PEGPH20) is a novel PEGylated recombinant human hyaluronidase developed as an anticancer therapy for use in combination with other systemic therapies to facilitate their delivery to the TME. Results from early clinical trials with PEGPH20 in patients with advanced solid tumors, including PC, were consistent with preclinical findings and supported additional clinical development [137, 138]. Unfortunately, the phase III HALO 109-301 clinical trial [139], which evaluates the efficacy and safety of PEGPH20 plus nab-paclitaxel/gemcitabine in patients with hyaluronan-high mPC, did not improve OS or PFS. However, it did show an increase in ORR (47% versus 36%). The safety profile of PEGPH20 was consistent with that found in previous studies. The study was well-conducted, with comparable progression rates and lower discontinuation rates than previous studies. However, the HALO-301 trial suggests the need to re-evaluate stroma remodeling strategies. PEGPH20 with modified FOLFIRINOX showed inferior survival, possibly due to dose reductions and unforeseen drug interactions. The complexity of tumor stroma and its role in PDA progression requires further investigation. Additionally, current methods for assessing hyaluronan status may be unreliable, and more data on metastatic sites are needed.

IACS-010759, a potent and selective OXPHOS inhibitor, was studied in phase I trials for relapsed/refractory acute myeloid leukemia (NCT02882321) and advanced solid tumors (NCT03291938) [140]. The drug had a narrow therapeutic index with dose-limiting toxicities, including elevated blood lactate and neurotoxicity, hindering target exposure. Modest target inhibition and limited antitumor activity were observed at tolerated doses, leading to trial discontinuation. Mouse studies showed IACS-010759 induced peripheral neuropathy, mitigated by coadministration of a HDAC6 inhibitor [141]. Further studies are needed to understand the link between OXPHOS inhibition and neurotoxicity, and caution is advised in developing complex I inhibitors as antitumor agents.

A phase Ib study in Japanese patients with advanced solid tumors investigated retifanlimab (anti-PD-1), INCB001158 (an oral ARG inhibitor), and their combination [142]. No fatal adverse events or discontinuations occurred. Retifanlimab showed a 33.3% overall response rate and 50% disease control rate. The combination treatment did not show additional benefit. Further research is needed to identify biomarkers predicting response to INCB001158 or the combination therapy.

Advances in metabolomics and transcriptomics are enabling the identification of tumor-specific metabolic vulnerabilities, paving the way for personalized metabolic interventions [105]. The integration of these technologies with existing immunotherapy strategies could improve patient stratification and therapeutic outcomes [106]. Among the possible strategies to overcome metabolic resistance to immunotherapy are:

1. **Combining metabolic inhibitors** with standard-of-care treatments, such as chemotherapy, radiotherapy, and ICIs, is a promising strategy to enhance therapeutic efficacy. However, optimizing dosing regimens and managing potential toxicities associated with metabolic interventions remain key challenges.
2. **Tumor microenvironment complexity**: The TME is highly dynamic and influenced by a variety of factors, including immune cell infiltration, hypoxia, and nutrient availability. A deeper understanding of the interplay between these factors and tumor metabolism is essential for the design of effective therapeutic strategies.
3. **Targeting metabolic plasticity**: Tumor cells exhibit remarkable metabolic plasticity, allowing them to switch between different metabolic pathways in response to environmental changes. Developing therapies that can target multiple metabolic pathways simultaneously or sequentially may be necessary to prevent resistance.
4. **Immunometabolism as a biomarker**: Emerging evidence suggests that metabolic markers, such as lactate levels and amino acid depletion, can serve as potential biomarkers for predicting response to immunotherapy. The development of reliable metabolic biomarkers could facilitate early detection of resistance and guide treatment adjustments.

## Conclusions

The interplay between metabolic pathways and immune evasion mechanisms within the TME is a critical factor contributing to resistance against ICI. Tumor cells exploit glycolysis to swiftly generate energy and adapt to the hypoxic and nutrient-deficient TME, while glutamine metabolism facilitates the synthesis of vital cellular components, including amino acids, nucleotides, and FAs. This metabolic reprogramming results in substantial alterations in the TME, characterized by the depletion of essential nutrients such as glucose and the accumulation of lactate, which together create an immunosuppressive milieu. Elevated lactate levels promote conditions that encourage the differentiation of macrophages into immunosuppressive M2-like phenotypes and recruit MDSCs, thereby diminishing the activity of lymphocytes and NK cells and impairing the efficacy of immunotherapy in solid tumors. Furthermore, the competition among macrophages for glucose intensifies this immunosuppression, underscoring the necessity for a more nuanced understanding of how nutrient consumption influences immune function.

FA accumulation represents another metabolic disruption that impacts various cell types within the TME, though the precise mechanisms remain to be fully elucidated. Given these complex metabolic dynamics, targeting specific pathways offers a promising approach to enhance immunotherapy outcomes. Recent preclinical studies have demonstrated the potential of metabolic co-immunotherapy, providing valuable insights into possible therapeutic interventions.

Despite the durable clinical responses achieved with ICIs in some cancer patients, resistance remains a significant challenge that limits the broader success of immunotherapy. Overcoming this barrier will likely require a multi-faceted approach, including novel clinical trials informed by mechanistic insights into ICI resistance, exploration of new immune targets, and integrating strategies that optimize the metabolic landscape of the TME. Standardized recommendations on dietary modifications, nutrient supplementation, and microbiota manipulation could also enhance ICI efficacy. Ultimately, selectively modulating metabolic pathways within the TME may not only improve immunotherapy response rates but also provide opportunities for synergistic combination treatments, paving the way for more effective cancer therapeutics in the future.

## Abbreviations

ARG-1: arginase-1
CAFs: cancer-associated fibroblasts
CTLs: cytotoxic T lymphocytes
ECM: extracellular matrix
FA: fatty acids
FAO: fatty acid oxidation
GLS: glutaminase
HIF: hypoxia-inducible factor
ICIs: immune checkpoint inhibitors
IDO1: inoleamine-2, 3-dioxygenase
IFN-γ: interferon gamma
mPC: metastatic pancreatic cancer
NFAT1: nuclear factor of activated T-cells 1
NHE1: Na/H exchanger isoform 1
NK: natural killer
ORR: objective response rate
OS: overall survival
OXPHOS: oxidative phosphorylation
PD-L1: programmed death-ligand 1
PFS: progression-free survival
RCC: renal clear-cell cancer
RIPK3: receptor-interacting protein kinase 3
TAMs: tumor-associated macrophages
TCA: tricarboxylic acid
TDO2: tryptophan-2, 3-dioxygenase
TME: tumor microenvironment

## Declarations

### Copyright

© The Author(s) 2025.
